# The Structure of Sum-Over-Paths, its Consequences, and Completeness for Clifford

**DOI:** 10.1007/978-3-030-71995-1_27

**Published:** 2021-03-23

**Authors:** Renaud Vilmart

**Affiliations:** 8 University of Oxford, Oxford, UK; 9 Sorbonne Université - LIP6, Paris, France; Université Paris-Saclay, ENS Paris-Saclay, Inria, CNRS, LMF, 91190 Gif-sur-Yvette, France

**Keywords:** Categorical Quantum Mechanics, Dagger-Compact PROP, Sum-Over-Paths, Clifford Fragment, Normal Form, Rewriting, Discard Construction, Verification

## Abstract

We show that the formalism of “Sum-Over-Path” (SOP), used for symbolically representing linear maps or quantum operators, together with a proper rewrite system, has the structure of a dagger-compact PROP. Several consequences arise from this observation:

– Morphisms of SOP are very close to the diagrams of the graphical calculus called ZH-Calculus, so we give a system of interpretation between the two

– A construction, called the discard construction, can be applied to enrich the formalism so that, in particular, it can represent the quantum measurement.

We also enrich the rewrite system so as to get the completeness of the Clifford fragments of both the initial formalism and its enriched version.

### 1 Introduction

The "Sum-Over-Paths" (SOP) formalism [1] was introduced in order to perform verification on quantum circuits. It is inspired by Feynman's notion of path-integrals, and can be conceived as a discrete version of it.

The core idea here is to represent unitary transformations in a symbolic way, so as to be able to simplify the term, which would for instance accelerate its evaluation. To do so, the formalism comes equipped with a rewrite system, which reduces any term into an equivalent one.

As pure quantum circuits (which represent unitary maps) can easily be mapped to an SOP morphism, one can try and perform verification: given a specification $\mathcal{S}$ and another SOP morphism $t$ obtained from a circuit supposed to implement the specification, we can compute the term $\mathcal{S}\circ t^\dagger$ and try to reduce it to the identity. In a very similar way, one can check whether two quantum circuits implement the same unitary map.

* This work was made during a Postdoc funded by the project PIA-GDN/Quantex. Proofs can be found at arXiv:2003.05678

The rewrite system is known to be complete for Clifford unitary maps, i.e. in the Clifford fragment of quantum mechanics, the term obtained from $t_1 \circ t_2^\dagger$ will reduce to the identity iff $t_1$ and $t_2$ represent the same unitary map. Moreover, this reduction terminates in time polynomial in the size of the SOP term (itself related to the size of the quantum circuit), and still performs well outside the Clifford fragment.

Lately, the SOP formalism has been used for efficient verification of optimisation strategies such as [4,12], as well as for specification of quantum circuits [6].

In this paper, we are interested in extensions of the formalism. We first focus on its categorical structure, and show that arbitrary terms already go beyond the representation of unitary maps. We then turn to extending the formalism to encompass mixed quantum processes. In both cases, we show a completeness result for their respective Clifford fragment.

In Section 2, we explain in details the structure of †-compact PROP, which we show in Section 3 to be shared by **SOP**.

Because the formalism is no longer restricted to unitary maps, we argue that it could benefit from a slight redefinition, which is done in Section 4.

Another "family" of categories that share this structure is the family of graphical languages for quantum computation: ZX-Calculus, ZW-Calculus and ZH-Calculus [3,7,8]. All three formalisms represent morphisms of **Qubit** using diagrams, and come with equational theories, proven to be complete for the whole category [3,11,19], i.e. whenever two diagrams represent the same morphism of **Qubit**, the first can be turned into the other using only the equational theory.

In Section 5, we present interpretations between the respective Clifford fragments of the ZH-calculus and **SOP**, in a slightly different way than in [14,15], partly thanks to our redefinition of sums-over-paths.

In Section 6, we realise that the original rewrite system of **SOP** is not enough for the completeness of the Clifford fragment of **Qubit**. We hence enrich the set of rules so as to get the completeness in this restriction.

In Section 7, we enrich the whole formalism using the discard construction [5], so as to be able to represent completely positive maps, as well as the operator of partial trace. Again, one can consider the Clifford fragment of this formalism. We give a new set of rewrite rules, and show that it makes the fragment complete.

### 2 Background

#### 2.1 PROPs and String Diagrams

The first kind of category we will be interested in is the *PROP* [13,20]. A PROP **C** is a strict symmetric monoidal category (SMC) [16,18] generated by a single object, or equivalently, whose objects form $\mathbb{N}$. Hence the morphisms of **C** are of the form $f : n \to m$. They can be composed sequentially $(. \circ .)$ or in parallel $(. \otimes .)$, and they satisfy the following axioms:

$$f \circ (g \circ h) = (f \circ g) \circ h \qquad f \otimes (g \otimes h) = (f \otimes g) \otimes h$$

$$id_m \circ f = f = f \circ id_n \qquad id_0 \otimes f = f = f \otimes id_0$$
$$(f_2 \circ f_1) \otimes (g_2 \circ g_1) = (f_2 \circ g_2) \circ (f_1 \otimes g_1)$$

The category is also equipped with a particular family of morphisms $\sigma_{n,m} : n + m \to m + n$. Intuitively, these allow morphisms to swap places. They satisfy additional axioms:

$$\sigma_{n,m+p} = (id_m \otimes \sigma_{n,p}) \circ (\sigma_{n,m} \otimes id_p) \qquad \sigma_{n+m,p} = (\sigma_{n,p} \otimes id_m) \circ (id_n \otimes \sigma_{m,p})$$
$$\sigma_{m,n} \circ \sigma_{n,m} = id_{n+m} \qquad (id_p \otimes f) \circ \sigma_{n,p} = \sigma_{m,p} \circ (f \otimes id_p)$$

### 2.2 †-Compact PROPs

Some PROPs can have additional structure, such as a compact-closed structure, or a †-functor.

A †-PROP **C** is a PROP together with an involutive, identity-on-objects functor $(.)^\dagger : \mathbf{C}^{\mathrm{op}} \to \mathbf{C}$ compatible with $(. \otimes .)$. That is, for every morphism $f : n \to m$, there is a morphism $f^\dagger : m \to n$ such that $f^{\dagger\dagger} = f$. It behaves with the compositions by $(f \circ g)^\dagger = g^\dagger \circ f^\dagger$ and $(f \otimes g)^\dagger = f^\dagger \otimes g^\dagger$. Finally, we have $\sigma_{n,m}^\dagger = \sigma_{m,n}$.

A †-compact PROP has two particular families of morphisms: $\eta_n : 0 \to 2n$ and $\epsilon_n : 2n \to 0$. These are dual by the †-functor: $\eta_n^\dagger = \epsilon_n$. They satisfy the following axioms:

$$(\epsilon_n \otimes id_n) \circ (id_n \otimes \eta_n) = id_n = (id_n \otimes \epsilon_n) \circ (\eta_n \otimes id_n)$$
$$\sigma_{n,n} \circ \eta_n = \eta_n \qquad \eta_{n+m} = (id_n \otimes \sigma_{n,m} \otimes id_m) \circ (\eta_n \otimes \eta_m)$$

In this context, one can define the transpose operator of a morphism $f$ as:

$$f^t := (\epsilon_m \otimes id_n) \circ (id_m \otimes f \otimes id_n) \circ (id_m \otimes \eta_m)$$

One can check that, thanks to the axioms of †-compact PROP, $(f \circ g)^t = g^t \circ f^t$, $(f \otimes g)^t = f^t \otimes g^t$, and $f^{tt} = f$.

We can then compose $(.)^t$ and $(.)^\dagger$: $\overline{(.)} := (.)^{\dagger t}$. Again using the axioms of †-compact PROP, one can check that $(.)^{\dagger t} = (.)^{t\dagger}$.

### 2.3 Example: Qubit

The usual example of a strict symmetric †-compact monoidal category is **FHilb**, the category whose objects are finite dimensional Hilbert spaces, and whose morphisms are linear maps between them. It is not, however, a PROP, as it is not generated by a single object.

One subcategory of **FHilb** that *is* a PROP, though, is **Qubit**, the subcategory of **FHilb** generated by the object $\mathbb{C}^2$, considered as the object 1. A morphism $f : n \to m$ of **Qubit** is hence a linear map from $\mathbb{C}^{2^n}$ to $\mathbb{C}^{2^m}$. $(. \circ .)$ is then the usual composition of linear maps, and $(. \otimes .)$ is the usual tensor product of linear maps. One can check that the first set of axioms is satisfied.

This is not enough to conclude that **Qubit** is a PROP. We still need to define a family of morphisms $\sigma_{n,m}$. In the Dirac notation, given a basis $\mathcal{B}$ of $\mathbb{C}^2$, we can define $\sigma_{n,m}$ as $\sigma_{n,m} := \sum\limits_{(\boldsymbol{x},\boldsymbol{y})\in\mathcal{B}^n\times\mathcal{B}^m} |\boldsymbol{y},\boldsymbol{x}\rangle\langle\boldsymbol{x},\boldsymbol{y}|$. One can then check that all the axioms of PROPs are satisfied.

**Qubit** is not only a PROP, but also †-compact. Indeed, first, given a morphism:

$$f = \sum_{(\boldsymbol{x},\boldsymbol{y})\in\mathcal{B}^n\times\mathcal{B}^m} a_{\boldsymbol{x},\boldsymbol{y}}\,|\boldsymbol{y}\rangle\langle\boldsymbol{x}|$$

we can define its dagger $f^\dagger := \sum\limits_{(\boldsymbol{x},\boldsymbol{y})\in\mathcal{B}^n\times\mathcal{B}^m} \overline{a_{\boldsymbol{x},\boldsymbol{y}}}\,|\boldsymbol{x}\rangle\langle\boldsymbol{y}|$, which is the usual definition of the dagger for linear maps.

Its compact structure can be given by $\eta_n := \sum\limits_{\boldsymbol{x}\in\mathcal{B}^n} |\boldsymbol{x},\boldsymbol{x}\rangle$, which implies $\epsilon_n = \eta_n^\dagger = \sum\limits_{\boldsymbol{x}\in\mathcal{B}^n} \langle\boldsymbol{x},\boldsymbol{x}|$. One can check that all the axioms of †-compact PROPs are satisfied.

Since **Qubit** is †-compact, we can define the transpose $(.)^t$ which happens to be the usual transpose of linear maps, and the conjugate $\overline{(.)}$, which again is the usual conjugation in linear maps over $\mathbb{C}$.

There is a subcategory of **Qubit** that is of importance: **Stab**. It is the smallest †-compact subcategory of **Qubit** (the compact structure is preserved) that contains:

- $|0\rangle : 0 \to 1$
- $H := \frac{1}{\sqrt{2}}(|0\rangle\langle 0| + |0\rangle\langle 1| + |1\rangle\langle 0| - |1\rangle\langle 1|) : 1 \to 1$
- $S := |0\rangle\langle 0| + i\,|1\rangle\langle 1| : 1 \to 1$
- $CZ := |00\rangle\langle 00| + |01\rangle\langle 01| + |10\rangle\langle 10| - |11\rangle\langle 11| : 2 \to 2$

## 3 The Category SOP

### 3.1 SOP as a PROP

The point of the Sum-Over-Paths formalism [1], is to *symbolically* manipulate morphisms written in a form akin to the Dirac notation. Reasoning on symbolic terms allow us to detect where a term can be simplified to a "smaller" one, or to give a specification on a term.

A morphism of the category will be of the form:
$|\boldsymbol{x}\rangle \mapsto s \sum\limits_{\boldsymbol{y}\in V^k} e^{2i\pi P(\boldsymbol{x},\boldsymbol{y})}\,|\boldsymbol{Q}(\boldsymbol{x},\boldsymbol{y})\rangle$ where:

- $\boldsymbol{x} = x_1, \ldots, x_n$ is the input signature, it is a list of variables
- $V$ is a set of variables (hence $\boldsymbol{y}$ is a collection of these variables)
- $P$ is a multivariate polynomial, instantiated by the variables $\boldsymbol{x}$ and $\boldsymbol{y}$
- $\boldsymbol{Q} = Q_1, \ldots, Q_m$ is the output signature, it is a multivariate, multivalued boolean polynomial
- $s$ is a real scalar

We may denote $V_f$ a subset of the variables $V$ used in $f$. Then by default, if $V_f$ and $V_g$ are used in the same term, we consider that $V_f \cap V_g = \varnothing$. To distinguish the two sum operators (the one in $P$ and the one in $\boldsymbol{Q}$), we can denote the one in the output signature $\boldsymbol{Q}$ as $\oplus$. Moreover, it will sometimes be necessary to immerse one of the boolean polynomials $Q_i$ in the polynomial $P$. We hence define $\widehat{Q_i}$ inductively as $\widehat{x} = x$ for a variable $x$, $\widehat{pq} = \widehat{p}\widehat{q}$ and $\widehat{p \oplus q} = \widehat{p} + \widehat{q} - 2\widehat{p}\widehat{q}$.

**Definition 1 (SOP). SOP** *is defined as the PROP where, given a set of variables $V$:*

- *Identity morphisms are $id_n : |\boldsymbol{x}\rangle \mapsto |\boldsymbol{x}\rangle$*
- *Morphisms $f : n \to m$ are of the form $f : |\boldsymbol{x}\rangle \mapsto s \sum\limits_{\boldsymbol{y} \in V^k} e^{2i\pi P(\boldsymbol{x},\boldsymbol{y})} |\boldsymbol{Q}(\boldsymbol{x},\boldsymbol{y})\rangle$*
  *where $s \in \mathbb{R}$, $\boldsymbol{x} \in V^n$, $P \in \mathbb{R}[X_1,\dots,X_{n+k}]/(1, X_i^2 - X_i)$,*
  *and $\boldsymbol{Q} \in (\mathbb{F}_2[X_1,\dots,X_{n+k}])^m$*
- *Composition is obtained as*
$$f \circ g := |\boldsymbol{x}_g\rangle \mapsto s_f s_g \sum_{\substack{\boldsymbol{y}_f \in V_f^{k_f} \\ \boldsymbol{y}_g \in V_g^{k_g}}} e^{2i\pi(P_g + P_f[\boldsymbol{x}_f \leftarrow \widehat{\boldsymbol{Q}_g}])} |\boldsymbol{Q}_f[\boldsymbol{x}_f \leftarrow \boldsymbol{Q}_g]\rangle$$
- *Tensor product is obtained as*
$$f \otimes g := |\boldsymbol{x}_f \boldsymbol{x}_g\rangle \mapsto s_f s_g \sum_{\substack{\boldsymbol{y}_f \in V_f^{k_f} \\ \boldsymbol{y}_g \in V_g^{k_g}}} e^{2i\pi(P_g + P_f)} |\boldsymbol{Q}_f \boldsymbol{Q}_g\rangle$$
- *The symmetric braiding is $\sigma_{n,m} : |\boldsymbol{x}_1, \boldsymbol{x}_2\rangle \mapsto |\boldsymbol{x}_2, \boldsymbol{x}_1\rangle$*

The polynomial $P$ is called the *phase polynomial*, as it appears in the morphism in $e^{2i\pi\cdot}$. Because of this, we consider the polynomial modulo 1. We also consider the polynomial quotiented by $X^2 - X$ for all its variables $X$, as these variables are to be evaluated in $\{0,1\}$, so we consider $X^2 = X$.

Notice that the definition of the identities does not directly fit the description of the morphisms. However, we can rewrite it as $|\boldsymbol{x}\rangle \mapsto |\boldsymbol{x}\rangle = |\boldsymbol{x}\rangle \mapsto 1 \sum\limits_{y \in V^0} e^{2i\pi 0} |\boldsymbol{x}\rangle$. Hence, when we sum over a single element, we may forget the sum operator, and when the phase polynomial is 0, we may not write it. Notice by the way that $id_0 = |\rangle \mapsto |\rangle$. Indeed, $|\rangle$ is absolutely valid, it represents an empty register.

*Example 1.* We can give the **SOP** version of the usual quantum gates:

$$R_Z(\alpha) := |x\rangle \mapsto e^{2i\pi \frac{\alpha x}{2\pi}} |x\rangle \qquad CNot := |x_1, x_2\rangle \mapsto |x_1, x_1 \oplus x_2\rangle$$
$$H := |x\rangle \mapsto \frac{1}{\sqrt{2}} \sum_{y \in V} e^{2i\pi \frac{xy}{2}} |y\rangle \qquad CZ := |x_1, x_2\rangle \mapsto e^{2i\pi \frac{x_1 x_2}{2}} |x_1, x_2\rangle$$

*Example 2.* Let us derive the operation $(id \otimes H) \circ CNot$:

$(id \otimes H) \circ CNot$

$$= \left( |x_1, x_2\rangle \mapsto \frac{1}{\sqrt{2}} \sum_{y \in V} e^{2i\pi \frac{x_2 y}{2}} |x_1, y\rangle \right) \circ \left( |x_1, x_2\rangle \mapsto |x_1, x_1 \oplus x_2\rangle \right)$$

$$= |x_1, x_2\rangle \mapsto \frac{1}{\sqrt{2}} \sum_{y \in V} e^{2i\pi \frac{(x_1 + x_2 - 2x_1 x_2)y}{2}} |x_1, y\rangle$$

where $x_1 + x_2 - 2x_1x_2 = \widehat{x_1 \oplus x_2}$.

The previous definition contains a claim: that **SOP** is a PROP. To be so, one has to check all the axioms of PROPs. One has to be careful when doing so. Indeed, the sequential composition $(. \circ .)$ induces a substitution. Hence, one has to check all the axioms in the presence of a "context", that is, one has to show that the axioms can be applied *locally*.

If an axiom states $t_1 \to t_2$, one should ideally check that $A \circ (id_n \otimes t_1 \otimes id_m) \circ B \to A \circ (id_n \otimes t_2 \otimes id_m) \circ B$ for any "before" morphism $B$ and any "after" morphism $A$. However, this can be easily reduced to checking that $A \circ t_1 \circ B \to A \circ t_2 \circ B$.

In the case of the axioms of PROPs, this can further be reduced to showing the axioms without context, as neither $id_n$ nor $\sigma_{n,m}$ introduce variables or phases. For the other axioms, however, the context will have to be taken into account. A fairly straightforward but tedious verification gives that, indeed, **SOP** is a PROP.

#### 3.2 From SOP to Qubit

To check the soundness of what we are going to do in the following, it may be interesting to have a way of interpreting morphisms of **SOP** as morphisms of **Qubit**.

**Definition 2.** *The functor* $[\![.]\!] : \mathbf{SOP} \to \mathbf{Qubit}$ *is defined as being identity on objects, and such that*

$$\left[\!\!\left[ |\boldsymbol{x}\rangle \mapsto s \sum_{\boldsymbol{y} \in V^k} e^{2i\pi P(\boldsymbol{x},\boldsymbol{y})} |Q(\boldsymbol{x},\boldsymbol{y})\rangle \right]\!\!\right] := s \sum_{(\boldsymbol{x},\boldsymbol{y}) \in \{0,1\}^n \times \{0,1\}^k} e^{2i\pi P(\boldsymbol{x},\boldsymbol{y})} |Q(\boldsymbol{x},\boldsymbol{y})\rangle\!\langle \boldsymbol{x}|$$

*Example 3.* The interpretation of $H$ is as intended the Hadamard gate:

$$[\![H]\!] = \frac{1}{\sqrt{2}} \sum_{x,y \in \{0,1\}} e^{2i\pi \frac{xy}{2}} |y\rangle\!\langle x| = \frac{1}{\sqrt{2}} \left( |0\rangle\!\langle 0| + |0\rangle\!\langle 1| + |1\rangle\!\langle 0| - |1\rangle\!\langle 1| \right)$$

**Proposition 1.** *The interpretation* $[\![.]\!]$ *is a* PROP-functor*, meaning:*
*i)* $[\![. \circ .]\!] = [\![.]\!] \circ [\![.]\!]$*,* *ii)* $[\![. \otimes .]\!] = [\![.]\!] \otimes [\![.]\!]$*,* *iii)* $[\![\sigma_{n,m}]\!] = \sigma_{n,m}$

#### 3.3 SOP as a †-Compact PROP

**Towards a Compact Structure.** It is tempting to try and adapt the compact structure of **Qubit** to **SOP**. To do so, we can first define $\eta_n := |\rangle \mapsto \sum_{\boldsymbol{y}\in V^n} |\boldsymbol{y},\boldsymbol{y}\rangle$. However, we cannot as easily define $\epsilon_n$. To do so, we need to put the phase polynomial to use: $\epsilon_n := |\boldsymbol{x}_1,\boldsymbol{x}_2\rangle \mapsto \frac{1}{2^n}\sum_{\boldsymbol{y}\in V^n} e^{2i\pi\frac{\boldsymbol{x}_1\cdot\boldsymbol{y}+\boldsymbol{x}_2\cdot\boldsymbol{y}}{2}}|\rangle$.

One can easily check that $[\![\epsilon_n]\!] = \epsilon_n$. We can also easily check that the axioms of †-compact PROP where $\epsilon_n$ does not appear, such as $\sigma_{n,n}\circ\eta_n = \eta_n$ and $(id_n\otimes\sigma_{n,m}\otimes id_m)\circ(\eta_n\otimes\eta_m) = \eta_{n+m}$ are satisfied.

However, the equation $(\epsilon_n\otimes id_n)\circ(id_n\otimes\eta_n) = id_n = (id_n\otimes\epsilon_n)\circ(\eta_n\otimes id_n)$ is not satisfied, as:

$$(\epsilon_n\otimes id_n)\circ(id_n\otimes\eta_n) = |\boldsymbol{x}\rangle \mapsto \frac{1}{2}\sum_{\boldsymbol{y}_1,\boldsymbol{y}_2\in V^n} e^{2i\pi\frac{\boldsymbol{x}\cdot\boldsymbol{y}_2+\boldsymbol{y}_1\cdot\boldsymbol{y}_2}{2}}|\boldsymbol{y}_1\rangle \neq id_n$$

The fact that we have $(\epsilon_n\otimes id_n)\circ(id_n\otimes\eta_n) \neq id_n$ while its interpretation in **Qubit** holds, hints at a way to *rewrite* the first term as the second.

**An Equational Theory.** A rewrite strategy is given in [1], and we show in Figure 1 the rules we are going to use in the paper. Each rewrite rule contains a condition, which usually ensures that a variable (the one we want to get rid of) does not appear in some polynomials. We hence use Var as the operator that gets all the variables from a sequence of polynomials. For simplicity, the input signature is omitted, as well as the parameters in the polynomials.

$$\sum_{\boldsymbol{y}} e^{2i\pi P}|\boldsymbol{Q}\rangle \underset{y_0\notin\mathrm{Var}(P,\boldsymbol{Q})}{\longrightarrow} 2\sum_{\boldsymbol{y}\setminus\{y_0\}} e^{2i\pi P}|\boldsymbol{Q}\rangle \tag{Elim}$$

$$\sum_{\boldsymbol{y}} e^{2i\pi\left(\frac{y_0}{2}(y_0'+\widehat{Q_2})+R\right)}|\boldsymbol{Q}\rangle \underset{\substack{y_0\notin\mathrm{Var}(R,Q_2,\boldsymbol{Q})\\ y_0'\notin\mathrm{Var}(Q_2)}}{\longrightarrow} 2\sum_{\boldsymbol{y}\setminus\{y_0,y_0'\}} e^{2i\pi\left(R[y_0'\leftarrow\widehat{Q_2}]\right)}\left|\boldsymbol{Q}\left[y_0'\leftarrow Q_2\right]\right\rangle \tag{HH}$$

$$\sum_{\boldsymbol{y}} e^{2i\pi\left(\frac{y_0}{4}+\frac{y_0}{2}\widehat{Q_2}+R\right)}|\boldsymbol{Q}\rangle \underset{y_0\notin\mathrm{Var}(Q_2,R,\boldsymbol{Q})}{\longrightarrow} \sqrt{2}\sum_{\boldsymbol{y}\setminus\{y_0\}} e^{2i\pi\left(\frac{1}{8}-\frac{1}{4}\widehat{Q_2}+R\right)}|\boldsymbol{Q}\rangle \tag{$\omega$}$$

**Fig. 1.** Rewrite strategy $\underset{\text{Clif}}{\longrightarrow}$.

$\underset{\text{Clif}}{\longrightarrow}$ denotes the rewrite system formed by the three rules (Elim), (HH) and $(\omega)$. $\overset{*}{\underset{\text{Clif}}{\longrightarrow}}$ is the transitive closure of the rewrite system. Notice that all the rules remove at least one variable from the morphism, so we know $\underset{\text{Clif}}{\longrightarrow}$ terminates.

When the rules are not oriented, we get an equivalence relation on the morphisms of **SOP**. We denote this equivalence $\underset{\text{Clif}}{\sim}$.

We denote $\mathbf{SOP}/\underset{\text{Clif}}{\sim}$ the category $\mathbf{SOP}$ quotiented by the equivalence relation $\underset{\text{Clif}}{\sim}$.

It is to be noticed that:

**Proposition 2.** *For any rule* $r$ *of* $\underset{\text{Clif}}{\longrightarrow}$ *and* $t_1, t_2 \in \mathbf{SOP}$*:*

$$t_1 \underset{r}{\longrightarrow} t_2 \implies \begin{cases} A \circ t_1 \circ B \underset{r}{\longrightarrow} A \circ t_2 \circ B & \textit{for all } A \textit{ and } B \textit{ composable} \\ A \otimes t_1 \otimes B \underset{r}{\longrightarrow} A \otimes t_2 \otimes B & \textit{for all } A \textit{ and } B \end{cases}$$

*This obviously generalises to* $\underset{\text{Clif}}{\sim}$.

This result allows us to forget about the context in the rewriting process.

The newly obtained category $\mathbf{SOP}/\underset{\text{Clif}}{\sim}$ is still a PROP. It even has a compact structure, as the last necessary axiom is now derivable:

$$(\epsilon \otimes id) \circ (id \otimes \eta) = |x\rangle \mapsto \frac{1}{2} \sum_{y_1, y_2 \in V} e^{2i\pi(\frac{y_1 y_2}{2} + \frac{x y_2}{2})} |y_1\rangle \underset{\text{(HH)}}{\longrightarrow} |x\rangle \mapsto |x\rangle = id$$

and similarly for $(id \otimes \epsilon) \circ (\eta \otimes id) = id$.

**†-Functor for SOP.** To show that $\mathbf{SOP}/\underset{\text{Clif}}{\sim}$ is †-compact, we lack a notion of †-functor $\mathbf{SOP}$.

Remember that we defined $\overline{(.)}$ as $(.)^{\dagger t}$. Since we have a compact structure, we can already define the functor $(.)^t$. Thanks to the new equivalence relation $\underset{\text{Clif}}{\sim}$, this functor is involutive. Hence, we have $(.)^\dagger = \overline{(.)}^t$. An appropriate definition of the conjugation can be given:

**Definition 3.** *The conjugation is defined as:*

$$\overline{|\boldsymbol{x}\rangle \mapsto s_f \sum e^{2i\pi P_f} |\boldsymbol{Q}_f\rangle} := |\boldsymbol{x}\rangle \mapsto s_f \sum e^{-2i\pi P_f} |\boldsymbol{Q}_f\rangle$$

By combination of $(.)^t$ this gives a definition of $(.)^\dagger$. These three functors are the expected ones:

**Proposition 3.** $[\![(.)^t]\!] = [\![.]\!]^t$ , $[\![\overline{(.)}]\!] = \overline{[\![.]\!]}$ , $[\![(.)^\dagger]\!] = [\![.]\!]^\dagger$

We can finally prove the wanted result:

**Theorem 1.** $\mathbf{SOP}/\underset{\text{Clif}}{\sim}$ *is a* †*-compact PROP.*

## 4 Redefinition of SOP

In **Qubit**, and hence in **SOP**, because the strutures are †-compact, it may feel unnatural to have an asymmetry between inputs and outputs of the process. Why not have morphisms of the form $f = s\sum_{\boldsymbol{y}} e^{2i\pi P}\,|\boldsymbol{O}\rangle\!\langle\boldsymbol{I}|$? In this case, we have to change the definition of the composition, which has for consequence that the **SOP** morphisms do not form a category. However, it is a category when quotiented by $\underset{\text{Clif}}{\sim}$. This is the reason why we did not define **SOP** like this at first, although it greatly simplifies the notions of compact structure and †-functor.

We now redefine **SOP**, and will use this new definition in the rest of the paper:

**Definition 4 (SOP).** *We redefine* **SOP** *as the collection of objects* $\mathbb{N}$ *and morphisms between them:*

- *Identity morphisms are* $id_n : \sum\limits_{\boldsymbol{y}\in V^n} |\boldsymbol{y}\rangle\!\langle\boldsymbol{y}|$
- *Morphisms* $f : n \to m$ *are of the form* $f : s \sum\limits_{\boldsymbol{y}\in V^k} e^{2i\pi P(\boldsymbol{y})}\,|\boldsymbol{O}(\boldsymbol{y})\rangle\!\langle\boldsymbol{I}(\boldsymbol{y})|$ *where* $s \in \mathbb{R}$, $P \in \mathbb{R}[X_1,\ldots,X_k]/(1, X_i^2 - X_i)$, $\boldsymbol{O} \in (\mathbb{F}_2[X_1,\ldots,X_k])^m$ *and* $\boldsymbol{I} \in (\mathbb{F}_2[X_1,\ldots,X_k])^n$
- *Composition is obtained as* $f\circ g := \frac{s_f s_g}{2^{|\boldsymbol{I}_f|}} \sum\limits_{\substack{\boldsymbol{y}_f,\boldsymbol{y}_g\\ \boldsymbol{y}\in V^m}} e^{2i\pi\left(P_g+P_f+\frac{\boldsymbol{O}_g\cdot\boldsymbol{y}+\boldsymbol{I}_f\cdot\boldsymbol{y}}{2}\right)}\,|\boldsymbol{O}_f\rangle\!\langle\boldsymbol{I}_g|$
- *Tensor product is obtained as* $f\otimes g := s_f s_g \sum\limits_{\boldsymbol{y}_f,\boldsymbol{y}_g} e^{2i\pi(P_g+P_f)}\,|\boldsymbol{O}_f\boldsymbol{O}_g\rangle\!\langle\boldsymbol{I}_f\boldsymbol{I}_g|$
- *The symmetric braiding is* $\sigma_{n,m} = \sum\limits_{\boldsymbol{y}_1,\boldsymbol{y}_2} |\boldsymbol{y}_2,\boldsymbol{y}_1\rangle\!\langle\boldsymbol{y}_1,\boldsymbol{y}_2|$
- *The compact structure is* $\eta_n = \sum\limits_{\boldsymbol{y}} |\boldsymbol{y},\boldsymbol{y}\rangle\!\langle|$ *and* $\epsilon_n = \sum\limits_{\boldsymbol{y}} |\rangle\!\langle\boldsymbol{y},\boldsymbol{y}|$
- *The †-functor is given by:* $f^\dagger := s\sum\limits_{\boldsymbol{y}} e^{-2i\pi P}\,|\boldsymbol{I}\rangle\!\langle\boldsymbol{O}|$
- *The functor* $[\![.]\!]$ *is defined as:* $[\![f]\!] := s \sum\limits_{\boldsymbol{y}\in\{0,1\}^k} e^{2i\pi P(\boldsymbol{y})}\,|\boldsymbol{O}(\boldsymbol{y})\rangle\!\langle\boldsymbol{I}(\boldsymbol{y})|$

As announced, this is not a category, as $id\circ id = \frac{1}{2}\sum_{\boldsymbol{y}} e^{2i\pi\frac{y_1+y_2}{2}y_3}\,|y_2\rangle\!\langle y_1| \neq \sum_y |y\rangle\!\langle y| = id$. This problem is solved by reintroducing the rewrite rules, adapted to the new formalism. In the following, references to the rewrite rules are to their adapted version.

The results given for the previous formalisation can easily be adapted. In particular:

**Proposition 4.** **SOP**/$\underset{\text{Clif}}{\sim}$ *is a †-compact PROP, and* $[\![.]\!]$ *is a †-compact PROP-functor.*

*Remark 1.* When building a **SOP**-morphism $t$ from a circuit (or a diagram as we will show in the following) in this formalism, provided the complexity of the gates is bounded (e.g. in the gateset $\langle H, R_Z(\alpha), CNot\,\rangle$), the resulting $t$ is always of size $O(d \times n)$ where $n$ is the size of the register, and $d$ the *depth* of the circuit (and for a diagram in $O(G \times a)$ where $G$ is the number of generators and $a$ the maximum arity of these generators). This contrasts with the first definition of **SOP**, where the size of the constructed **SOP** term gets exponential in general.

### 5 SOP and Graphical Languages

The sum-over-paths formalism was initially intended to be used for isometries. As such, it was given a weak form of completeness – as we will discuss in the next section. However, if transforming a quantum circuit – that describes an isometry – into an **SOP** morphism is easy, the converse, transforming a **SOP** morphism into a circuit is not. And actually, all **SOP** morphisms do not represent an isometry. For instance, the morphism $\epsilon_1$ described above is not an isometry. An even smaller example is $\sum_y |\rangle\langle y|$ which is a valid **SOP** morphism, but clearly does not represent an isometry.

Monoidal categories, and subsequently PROPs, have the benefit of having a nice graphical representation, using string diagrams. The fact that **SOP** is one hints at another (family) of language(s) more suited for representing it: the Z$*$-Calculi: ZX, ZW and ZH [7,8,10,3]. These are all †-compact graphical languages, that have an interpretation in **Qubit**, and are universal for **Qubit**. This means that any morphism of **Qubit** can be represented as a morphism of either of these 3 languages.

The language that happens to be the closest to **SOP** is the ZH-Calculus. This is the one we are going to present in the following. However, bear in mind that, as we have semantics-preserving functors between any two of these three languages, one can do the same work with ZX and ZW-Calculi.

The link between the sum-over-paths formalism and the ZH-Calculus was first shown in [14,15]. We give here a slightly different but equivalent presentation, that in particular uses the fact that we altered the formalism of **SOP**, and we will focus this presentation to the Clifford fragment, as it is sufficient for the scope of the present article, although a more general presentation could be given (see the previous two references, or the longer version of the present article).

#### 5.1 The Cliffrord Fragment of the ZH-Calculus

$\mathbf{ZH}_{\text{Clif}}$ is a PROP whose morphisms are composed (sequentially $(. \circ .)$ or in parallel $(. \otimes .)$) from the generators  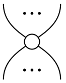 ,  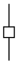 ,  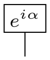 and $\boxed{s}$ ; where $\alpha \in \frac{\pi}{2}\mathbb{Z}$ and $s \in \langle\sqrt{2}, e^{i\frac{\pi}{4}}\rangle$ the multiplicative group freely generated by $\sqrt{2}$ and $e^{i\frac{\pi}{4}}$.

$\mathbf{ZH}_{\text{Clif}}$ is made a †-compact PROP, which means it also has the symmetric structure $\sigma_{n,m}$ :: 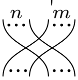 , the compact structure $\left(\eta_n :: \right.$ 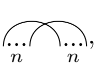 , $\epsilon_n ::$ 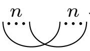 $\left.\right)$, and a †-functor $(.)^\dagger : \mathbf{ZH}_{\text{Clif}}^{\text{op}} \to \mathbf{ZH}_{\text{Clif}}$.

For convenience, we define two additional spiders:

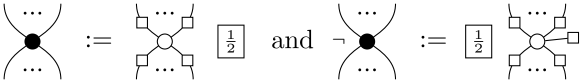

The full language comes with a way of interpreting the morphisms as morphisms of **Qubit**, and whose restriction to $\mathbf{ZH}_{\mathrm{Clif}}$ maps to **Stab**. The standard interpretation $[\![.]\!] : \mathbf{ZH}_{\mathrm{Clif}} \to \mathbf{Stab}$ is a †-compact-PROP-functor, defined as:

$[\![$  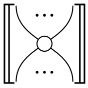 $]\!] = |0^m\rangle\langle 0^n| + |1^m\rangle\langle 1^n|$, $[\![$  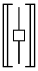 $]\!] = \sum_{x,y\in\{0,1\}} (-1)^{xy} |y\rangle\langle x|$,

$[\![$  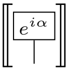 $]\!] = |0\rangle + e^{i\alpha} |1\rangle$, $[\![$  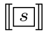 $]\!] = s$

Notice that we used the same symbol for two different functors: the two interpretations $[\![.]\!] : \mathbf{SOP} \to \mathbf{Qubit}$ and $[\![.]\!] : \mathbf{ZH}_{\mathrm{Clif}} \to \mathbf{Stab}$. It should be clear from the context which one is to be used.

The language is universal for **Stab**:

**Proposition 5.** $[\![.]\!] : \mathbf{ZH}_{\mathrm{Clif}} \to \mathbf{Stab}$ *is onto, i.e.*

$$\forall f \in \mathbf{Stab},\ \exists D_f \in \mathbf{ZH}_{\mathrm{Clif}},\quad [\![D_f]\!] = f$$

Since it is not a 1-to-1 correspondence, the language comes with an equational theory, which in particular gives the axioms for a †-compact PROP. We will not present it here.

### 5.2 From $\mathbf{ZH}_{\mathrm{Clif}}$ to SOP

We show in this section how any $\mathbf{ZH}_{\mathrm{Clif}}$ morphism can be turned into a **SOP** morphism in a way that preserves the semantics. We define $[.]^{\mathrm{sop}} : \mathbf{ZH}_{\mathrm{Clif}} \to \mathbf{SOP}$ as the †-compact PROP-functor such that:

$[$  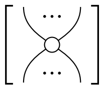 $]^{\mathrm{sop}} := \sum_{y} |y,\dots,y\rangle\langle y,\dots,y|$ $[$  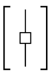 $]^{\mathrm{sop}} := \sum_{y_0,y_1} e^{2i\pi\frac{y_0y_1}{2}} |y_0\rangle\langle y_1|$

$[$  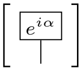 $]^{\mathrm{sop}} := \sum_{y} e^{2i\pi\frac{\alpha}{2\pi}y} |y\rangle$ $[$  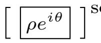 $]^{\mathrm{sop}} := \rho\sum_{\emptyset} e^{2i\pi\frac{\theta}{2\pi}} |\rangle\langle|$ for $\rho e^{i\theta}\in\langle\sqrt{2},e^{i\frac{\pi}{4}}\rangle$

This interpretation can be extended to the full graphical language. It preserves the semantics:

**Proposition 6.** $[\![[.]^{\mathrm{sop}}]\!] = [\![.]\!]$.

### 5.3 The Clifford Fragment of SOP

Since $\mathbf{ZH}_{\mathrm{Clif}}$ is universal for **Stab**, the Clifford fragment of **Qubit**, and since we have an interpretation $[.]^{\mathrm{sop}} : \mathbf{ZH}_{\mathrm{Clif}} \to \mathbf{SOP}$ that preserves the semantics, we can define $\mathbf{SOP}_{\mathrm{Clif}}$ as the the image of $\mathbf{ZH}_{\mathrm{Clif}}$ by $[\![.]\!]$. This gives a characterisation of the fragment:

**Definition 5.** $\mathbf{SOP}_{\text{Clif}}$ *is the subPROP of* **SOP** *with the same objects, and whose morphisms are of the form* $\frac{1}{\sqrt{2}^p}\sum e^{2i\pi\left(\frac{1}{8}P^{(0)}+\frac{1}{4}P^{(1)}+\frac{1}{2}P^{(2)}\right)}\left|\boldsymbol{O}\middle\rangle\!\middle\langle\boldsymbol{I}\right|$ *where* $P^{(i)}$ *is a polynomial with integer coefficients of degree at most* $i$ *(hence* $P^{(0)}$ *is in fact merely an integer); and where all the* $O_i$ *and* $I_i$ *are linear.*

It is an easy check that $[\mathbf{ZH}_{\text{Clif}}]^{\text{sop}} \subseteq \mathbf{SOP}_{\text{Clif}}$, so $\mathbf{SOP}_{\text{Clif}}$ has enough morphisms to describe the Clifford fragment of quantum computing. We can even show it exactly captures it. To do so, we introduce an interpretation from $\mathbf{SOP}_{\text{Clif}}$ back to $\mathbf{ZH}_{\text{Clif}}$.

#### 5.4 From $\mathbf{SOP}_{\text{Clif}}$ to $\mathbf{ZH}_{\text{Clif}}$

We define $[.]^{\text{ZH}} : \mathbf{SOP}_{\text{Clif}} \to \mathbf{ZH}_{\text{Clif}}$ on arbitrary $\mathbf{SOP}_{\text{Clif}}$ morphisms as:

$$\left[s\sum_{\boldsymbol{y}} e^{2i\pi P}\left|O_1,\ldots,O_m\middle\rangle\!\middle\langle I_1,\ldots,I_n\right|\right]^{\text{ZH}} :=$$

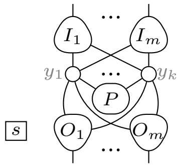

where the row of Z-spiders represents the variables $y_1,\ldots,y_k$.

The inputs of $O_i$ are linked to $y_1,\ldots,y_k$. The nodes $O_i$ can be inductively defined as:

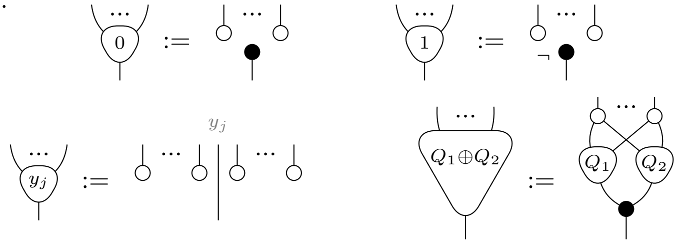

Notice that we did not define how to interpret a product $Q_1Q_2$. This can be done for the interpretation of the full **SOP** category, but it is unnecessary for $\mathbf{SOP}_{\text{Clif}}$ where the $O_i$ are linear. The nodes $I_i$ are defined similarly, but upside-down. The node $P$ can be inductively defined as:

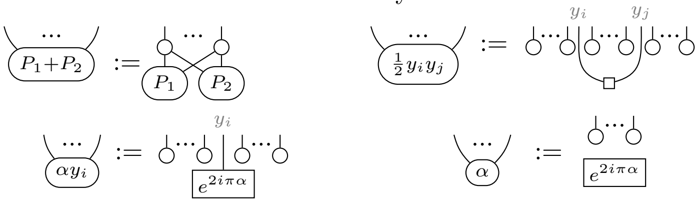

The obtained diagram can then be reduced using usual rules of **ZH**.

The system of interpretations is close to preserving the structure of the terms:

**Proposition 7.** $\left[[.]^{\text{ZH}}\right]^{\text{sop}} \underset{\text{Clif}}{\sim} (.)$

**Corollary 1.** $[\![[.]^{\text{ZH}}]\!] = [\![.]\!]$.

This result allows us to prove $\mathbf{SOP}_{\text{Clif}}$ does capture the Clifford fragment of quantum mechanics:

**Proposition 8.** $[\![.]\!] : \mathbf{SOP}_{\text{Clif}} \to \mathbf{Stab}$*, the restriction of the standard interpretation to* $\mathbf{SOP}_{\text{Clif}}$ *is onto* $\mathbf{Stab}$*.*

### 6 A Complete Rewrite System for Clifford

In [1], where the rewrite rules are introduced, the author gives a notion of completeness for Clifford *unitaries*, that we will refer to in the following as "weak completeness":

**Proposition 9 (Weak Completeness for Clifford Unitaries).** *Given two terms* $t_1$*,* $t_2$ *of* $\mathbf{SOP}_{\text{Clif}}$ *such that* $[\![t_i]\!] \circ [\![t_i]\!]^\dagger = id = [\![t_i]\!]^\dagger \circ [\![t_i]\!]$*, we have:*

$$t_1 \circ t_2^\dagger \underset{\text{Clif}}{\overset{*}{\longrightarrow}} id \quad \iff \quad [\![t_1]\!] = [\![t_2]\!]$$

In practice, this is sufficient for deciding the equivalence of two Clifford quantum circuits, as they are represented as unitary morphisms of $\mathbf{SOP}_{\text{Clif}}$. However, in our case, where we deal with more than unitaries, we cannot use this trick. Instead, we aim at a result like "$t_1 \overset{*}{\longrightarrow} t \overset{*}{\longleftarrow} t_2 \iff [\![t_1]\!] = [\![t_2]\!]$". In other words, we want a rewrite system that will transform any term of $\mathbf{SOP}_{\text{Clif}}$ into a unique normal form. However, the rewrite system $\underset{\text{Clif}}{\longrightarrow}$ is not enough for this:

**Lemma 1.** $\underset{\text{Clif}}{\longrightarrow}$ *is not confluent in* $\mathbf{SOP}_{\text{Clif}}$*.*

To address this problem, we propose to add three rewrite rules to the previously presented ones. These new rewrite rules are shown in Figure 2.

$$\sum e^{2i\pi(P)} |O_1, ..., \underbrace{y_0 \oplus O_i'}_{O_i}, ..., O_m\rangle\langle \boldsymbol{I}| \underset{y_0 \notin \text{Var}(O_1,...,O_{i-1},O_i') \,\wedge\, O_i' \neq 0}{\longrightarrow} \sum e^{2i\pi\left(P[y_0 \leftarrow \widehat{O_i}]\right)} (|\boldsymbol{O}\rangle\langle \boldsymbol{I}|)[y_0 \leftarrow O_i] \qquad \text{(ket)}$$

$$\sum e^{2i\pi(P)} |\boldsymbol{O}\rangle\langle I_1, ..., \underbrace{y_0 \oplus I_i'}_{I_i}, ..., I_m| \underset{y_0 \notin \text{Var}(\boldsymbol{O},I_1,...,I_{i-1},I_i') \,\wedge\, I_i' \neq 0}{\longrightarrow} \sum e^{2i\pi\left(P[y_0 \leftarrow \widehat{I_i}]\right)} (|\boldsymbol{O}\rangle\langle \boldsymbol{I}|)[y_0 \leftarrow I_i] \qquad \text{(bra)}$$

$$s\sum_{\boldsymbol{y}} e^{2i\pi\left(\frac{y_0}{2}+R\right)} |\boldsymbol{O}\rangle\langle \boldsymbol{I}| \underset{(R\neq 0 \text{ or } \boldsymbol{OI}\neq\boldsymbol{0}) \,\wedge\, y_0 \notin \text{Var}(R,\boldsymbol{O},\boldsymbol{I})}{\longrightarrow} \sum_{y_0} e^{2i\pi\left(\frac{y_0}{2}\right)} |0, ..., 0\rangle\langle 0, ..., 0| \qquad \text{(Z)}$$

**Fig. 2.** Together with those of $\underset{\text{Clif}}{\longrightarrow}$, these rules constitute the rewrite system $\underset{\text{Clif+}}{\longrightarrow}$.

The last rule (Z) describes what happens for a term that represents the linear map 0. Rule (bra) is simply the continuation of (ket). They explain how to operate suitable changes of variables.

**Proposition 10.** *The rewrite system* $\underset{\text{Clif+}}{\longrightarrow}$ *terminates.*

Not only does this rewrite system terminate, it is confluent in $\mathbf{SOP}_{\text{Clif}}$ and the induced equivalence relation $\underset{\text{Clif+}}{\sim}$ is complete for Clifford. The plan to prove this is by showing that any morphism of $\mathbf{SOP}_{\text{Clif}}$ reduces to a normal form that is unique, up to $\alpha$-conversion (upcoming Thm. 2). To get there, we first need a few intermediary results.

**Lemma 2.** *Any morphism of* $\mathbf{SOP}_{\text{Clif}}$ *reduces by* $\underset{\text{Clif+}}{\longrightarrow}$ *to a morphism of the form* $\frac{1}{\sqrt{2}^p}\sum e^{2i\pi P}\,|\boldsymbol{O}\rangle\!\langle\boldsymbol{I}|$ *where:*

- $\text{Var}(P) \subseteq \text{Var}(\boldsymbol{O},\boldsymbol{I})$ *or* $P = \frac{y_0}{2}$ *where* $y_0 \notin \text{Var}(\boldsymbol{O},\boldsymbol{I})$
- $O_i = \begin{cases}\text{either } y_k \text{ or} \\ c \oplus \bigoplus\limits_{y\in\text{Var}(O_1,\dots,O_{i-1})} c_y y & \textit{where } c, c_y \in \{0,1\}\end{cases}$
- $I_i = \begin{cases}\text{either } y_k \text{ or} \\ c \oplus \bigoplus\limits_{y\in\text{Var}(\boldsymbol{O},I_1,\dots,I_{i-1})} c_y y & \textit{where } c, c_y \in \{0,1\}\end{cases}$

To start with, we deal with the case where the term represents the null map.

**Proposition 11.** *Let* $t$ *be a morphism of* $\mathbf{SOP}_{\text{Clif}}$ *such that* $[\![t]\!] = 0$. *Then:*

$$t \underset{\text{Clif+}}{\overset{*}{\longrightarrow}} \sum_{y_0} e^{2i\pi\frac{y_0}{2}}\,|0,\dots,0\rangle\!\langle 0,\dots,0|$$

**Corollary 2.** *If a morphism* $t = \frac{1}{\sqrt{2}^p}\sum e^{2i\pi P}\,|\boldsymbol{O}\rangle\!\langle\boldsymbol{I}|$ *of* $\mathbf{SOP}_{\text{Clif}}$ *is irreducible such that* $\text{Var}(P) \subseteq \text{Var}(\boldsymbol{O},\boldsymbol{I})$, *then* $[\![t]\!] \neq 0$.

Before moving on to the completeness by normal forms theorem, we need a result for the uniqueness of the phase polynomial:

**Lemma 3.** *Let* $P_1$ *and* $P_2$ *be two polynomials of* $\mathbb{R}[X_1,\dots,X_k]/(1, X^2 - X)$. *We have* $\left(\forall \boldsymbol{x} \in \{0,1\}^k,\ P_1(\boldsymbol{x}) = P_2(\boldsymbol{x})\right) \implies (P_1 = P_2)$

**Theorem 2.** *Let* $t_1$, *and* $t_2$ *be two morphisms of* $\mathbf{SOP}_{\text{Clif}}$ *such that* $[\![t_1]\!] = [\![t_2]\!]$. *Then, there exists* $t$ *in* $\mathbf{SOP}_{\text{Clif}}$ *such that* $t_1 \underset{\text{Clif+}}{\overset{*}{\longrightarrow}} t \underset{\text{Clif+}}{\overset{*}{\longleftarrow}} t_2$, *up to* $\alpha$*-conversion.*

This result is not totally surprising, since, as exposed by [15], the rules of $\underset{\text{Clif}}{\longrightarrow}$ are generalisations of the so-called pivoting and local complementation which can be used to reduce any Clifford ZX (or ZH)-diagram into a *pseudo*-normal form [9,2] there, a diagram can have several different but equivalent pseudo-normal form. The rules introduced to get $\underset{\text{Clif+}}{\longrightarrow}$ are simply here to further rewrite terms in pseudo-normal form into terms in proper (unique) normal form.

**Corollary 3.** *The equality of morphisms in* $\mathbf{SOP}_{\mathrm{Clif}}/\underset{\mathrm{Clif+}}{\sim}$ *is decidable in time polynomial in the size of the phase polynomial and in the combined size of the ket/bra polynomials.*

Although the set of rules is confluent in $\mathbf{SOP}_{\mathrm{Clif}}$, it is not in $\mathbf{SOP}$:

**Lemma 4 (Non-confluence).** *The rewrite systems* $\underset{\mathrm{Clif}}{\longrightarrow}$ *and* $\underset{\mathrm{Clif+}}{\longrightarrow}$ *are not confluent in* $\mathbf{SOP}$.

### 7 SOP with Discards

We want in this section to extend **SOP** to be able to express the larger formalism of mixed quantum operators. The discard construction can be used for that purpose, as well as for extending the rewrite system for the Clifford fragment. We finally leverage the previous completeness theorem to get a similar result in this extension.

#### 7.1 The Discard Construction on SOP

In [5], a construction is given to extend any †-compact PROP for *pure* quantum mechanics to another †-compact PROP for quantum mechanics with environment. This new formalism can also be understood as the previous one, but where on top of it, one can discard the qubits. Because **SOP** fits the requirements, the construction can be applied to it.

First, we have to create the subcategory $\mathbf{SOP}_{\mathrm{iso}}$ of **SOP** that contains all its isometries. The objects of the new category are the same, and its morphisms are $\{f \in \mathbf{SOP} \mid [\![f^\dagger \circ f]\!] = id\}$.

These are important, as the isometries are exactly the pure quantum operators that can be discarded. The next step in the construction does just that. We perform the affine completion of $\mathbf{SOP}_{\mathrm{iso}}$, that is, for every object $n$, we add a new morphism $!_n : n \to 0$, and we impose that $! \circ f =!$ for any $f$ in the new category, that we denote $\mathbf{SOP}_{\mathrm{iso}}^!$. We also need to impose that $!_n \otimes !_m =!_{n+m}$ and $!_0 = id_0$.

Finally, the category $\mathbf{SOP}^{\bar{\top}}$ is obtained as the following pushout in the category of SMCs, where the arrows are the inclusion functors:

$$\begin{array}{ccc} \mathbf{SOP}_{\mathrm{iso}} & \longrightarrow & \mathbf{SOP} \\ \downarrow & & \downarrow \\ \mathbf{SOP}_{\mathrm{iso}}^! & \longrightarrow & \mathbf{SOP}^{\bar{\top}} \end{array}$$

We write the new morphisms in the form $s \sum_{\boldsymbol{y}\in V^k} e^{2i\pi P(\boldsymbol{y})} \left|\boldsymbol{O}(\boldsymbol{y})\right\rangle !\boldsymbol{D}(\boldsymbol{y}) \left\langle \boldsymbol{I}(\boldsymbol{y})\right|$ where the additional $\boldsymbol{D}$ is a set of multivariate polynomials of $\mathbb{F}_2$. The fact that it is a set, and not a list, already captures some rules on the discard: first permuting qubits and then discarding them is equivalent to discarding them right away. Similarly, copying data and discarding the copies is equivalent to discarding the data right away.

Pure morphisms are those such that $\boldsymbol{D} = \{\}$. In those, no qubits are discarded. We hence easily induce usual morphisms such as $H$ and $CZ$ in the new formalism.

The new morphisms $!_n$ are given by: $!_n := \sum_{\boldsymbol{y}\in V^n} |\rangle !\{y_1,\dots,y_n\}\langle y_1,\dots,y_n|$

In the new formalism, the compositions are obtained exactly like previously, where the resulting set of discarded polynomial is the union of the other two.

It might be useful to be able to give an interpretation to the morphisms of the new formalism. To do so, we use the CPM construction [17] to map morphisms of $\mathbf{SOP}^{\text{⏚}}$ to morphisms of $\mathbf{SOP}$.

**Definition 6.** *The map* CPM : $\mathbf{SOP}^{\text{⏚}} \to \mathbf{SOP}$ *is defined as:*

$$s\sum_{\boldsymbol{y}} e^{2i\pi P} |\boldsymbol{O}\rangle !\boldsymbol{D}\langle \boldsymbol{I}| \;\mapsto$$
$$\frac{s^2}{2^{|\boldsymbol{D}|}} \sum_{\boldsymbol{y}_1,\boldsymbol{y}_2,\boldsymbol{y}} e^{2i\pi\left(P(\boldsymbol{y}_1)-P(\boldsymbol{y}_2)+\frac{\boldsymbol{D}(\boldsymbol{y}_1)\cdot\boldsymbol{y}+\boldsymbol{D}(\boldsymbol{y}_2)\cdot\boldsymbol{y}}{2}\right)} |\boldsymbol{O}(\boldsymbol{y}_1),\boldsymbol{O}(\boldsymbol{y}_2)\rangle\langle \boldsymbol{I}(\boldsymbol{y}_1),\boldsymbol{I}(\boldsymbol{y}_2)|$$

We can now define a standard interpretation of $\mathbf{SOP}^{\text{⏚}}$-morphisms as:

**Definition 7.** *The standard interpretation* $[\![.]\!]$ *of* $\mathbf{SOP}^{\text{⏚}}$ *is defined as* $[\![.]\!] := [\![\mathrm{CPM}(.)]\!]$.

Again, it is easy to transform any morphism of $\mathbf{SOP}^{\text{⏚}}$ in $\mathbf{ZH}^{\text{⏚}}$ and vice-versa:

$$\left[ s\sum_{\boldsymbol{y}\in V^k} e^{2i\pi P(\boldsymbol{y})} |\boldsymbol{O}(\boldsymbol{y})\rangle !\boldsymbol{D}(\boldsymbol{y})\langle \boldsymbol{I}(\boldsymbol{y})| \right]^{\mathrm{ZH}} :=$$

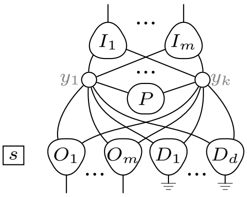

and $[\text{⏚}]^{\mathrm{sop}} = !_1$.

#### 7.2 SOP with Discards for Clifford

The discard construction can be applied to the subcategory $\mathbf{SOP}_{\mathrm{Clif}}$. We end up with a new category $\mathbf{SOP}^{\text{⏚}}_{\mathrm{Clif}}$, such that the following diagram, whose arrows are inclusions, commutes:

$$\begin{array}{ccc} \mathbf{SOP}_{\mathrm{Clif}} & \longrightarrow & \mathbf{SOP} \\ \downarrow & & \downarrow \\ \mathbf{SOP}^{\text{⏚}}_{\mathrm{Clif}} & \longrightarrow & \mathbf{SOP}^{\text{⏚}} \end{array}$$

Following the characterisation of $\mathbf{SOP}_{\mathrm{Clif}}$ morphisms, we determine that all the morphisms of $\mathbf{SOP}^{\text{⏚}}_{\mathrm{Clif}}$ are of the form: $\frac{1}{\sqrt{2}^p}\sum e^{2i\pi\left(\frac{1}{8}P^{(0)}+\frac{1}{4}P^{(1)}+\frac{1}{2}P^{(2)}\right)} |\boldsymbol{O}\rangle !\boldsymbol{D}\langle \boldsymbol{I}|$ where $p \in \mathbb{Z}$, where $P^{(i)}$ is a polynomial with integer coefficients and of degree at most $i$, and where the polynomials of $\boldsymbol{O}, \boldsymbol{D}$ and $\boldsymbol{I}$ are linear.

The rewrite system presented previously can obviously be adapted to the new formalism (when there is a substitution, it has to be applied in $!\boldsymbol{D}$ as well).

On top of that, the condition that makes $\mathbf{SOP}^{!}_{\text{iso}}$ terminal can be translated as a meta rule which sadly is not easy to apply. Thankfully, the last part of [5] is devoted to showing that this big meta rule can sometimes be replaced by a few small ones. The idea is that, in some cases (in particular in the Clifford fragment), all the isometries can be generated from a finite set of generators. In particular, it is enough to impose the following equations:

$$e^{i\alpha} = 1 \qquad !_1 \circ |0\rangle = 1 \qquad !_1 \circ H = ! \qquad !_1 \circ S = !_1 \qquad !_2 \circ CZ = !_2$$

Based on this, we can give an updated set of rewrite rules fit for the introduction of $\stackrel{\perp}{=}$. Due to the size of this rewrite system, we do not provide it here, but it can be found in the extended version of this paper. The rewrite system is denoted $\underset{\text{Clif}\,\stackrel{\perp}{=}}{\longrightarrow}$ and induces a equivalence relation $\underset{\text{Clif}\,\stackrel{\perp}{=}}{\sim}$. Notice that we can extend CPM to $\text{CPM} : \mathbf{SOP}^{\stackrel{\perp}{=}}/\underset{\text{Clif}\,\stackrel{\perp}{=}}{\sim} \to \mathbf{SOP}/\underset{\text{Clif+}}{\sim}$, which makes it a functor.

**Proposition 12.** *The rewrite system* $\underset{\text{Clif}\,\stackrel{\perp}{=}}{\longrightarrow}$ *terminates.*

We aim to prove a similar result to that of the $\stackrel{\perp}{=}$-free Clifford fragment, that is that the new rewrite system rewrites any morphism of the Clifford fragment into a unique normal form. The idea here it to make use of the previous result.

**Lemma 5.** *Any non-null morphism of* $\mathbf{SOP}^{\stackrel{\perp}{=}}_{\text{Clif}}$ *can be reduced to:*

$$\frac{1}{\sqrt{2}^p}\sum_{\boldsymbol{y},\boldsymbol{y}_d} e^{2i\pi\left(\frac{1}{4}P^{(1)}(\boldsymbol{y})+\frac{1}{2}P^{(2)}(\boldsymbol{y},\boldsymbol{y}_d)\right)} |\boldsymbol{O}(\boldsymbol{y},\boldsymbol{y}_d)\rangle!\{\boldsymbol{y}_d\}\,\langle\boldsymbol{I}(\boldsymbol{y},\boldsymbol{y}_d)| \quad \textit{where:}$$

- *polynomials of* $\boldsymbol{O}$ *and* $\boldsymbol{I}$ *are linear*
- *the set of discarded polynomials is reduced to a set of variables* $\{\boldsymbol{y}_d\}$
- $P^{(1)}$ *and* $P^{(2)}$ *have no constants*
- *no monomial of* $P^{(2)}$ *uses only variables of* $\boldsymbol{y}_d$
- $\{\boldsymbol{y}_d\} \subseteq \text{Var}(\boldsymbol{O},\boldsymbol{I})$
- $\text{Var}(P^{(1)},P^{(2)}) \subseteq \text{Var}(\boldsymbol{O},\boldsymbol{I},\boldsymbol{D})$ *or* $P = \frac{y_0}{2}$ *with* $y_0 \notin \text{Var}(\boldsymbol{O},\boldsymbol{I},\boldsymbol{D})$.

**Corollary 4.** *Any morphism of* $\mathbf{SOP}^{\stackrel{\perp}{=}}_{\text{Clif}}$ *eventually reduces to a morphism of the form given in Lem. 5.*

**Lemma 6.** *Any morphism* $t$ *of* $\mathbf{SOP}^{\stackrel{\perp}{=}}_{\text{Clif}}$ *such that* $[\![t]\!] = 0$ *reduces to:*

$$\sum_{y_0} e^{2i\pi\left(\frac{y_0}{2}\right)} |0,\cdots,0\rangle!\{\}\,\langle 0,\cdots,0|$$

**Corollary 5.** *If* $t \in \mathbf{SOP}^{\stackrel{\perp}{=}}_{\text{Clif}}$ *is terminal with* $\text{Var}(P) \subseteq \text{Var}(\boldsymbol{O},\boldsymbol{D},\boldsymbol{I})$, *then* $[\![t]\!] \neq 0$.

**Definition 8.** *We define* $\overline{\mathbf{SOP}^{\stackrel{\perp}{=}}_{\text{Clif}}}$ *as the set of morphisms of* $\mathbf{SOP}^{\stackrel{\perp}{=}}_{\text{Clif}}$ *in the form given in Lem. 5. We define the function* $F$ *on* $\overline{\mathbf{SOP}^{\stackrel{\perp}{=}}_{\text{Clif}}}$ *such that, for any morphism* $t = \frac{1}{\sqrt{2}^p}\sum_{\boldsymbol{y},\boldsymbol{y}_d} e^{2i\pi P(\boldsymbol{y},\boldsymbol{y}_d)} |\boldsymbol{O}(\boldsymbol{y},\boldsymbol{y}_d)\rangle!\{\boldsymbol{y}_d\}\,\langle\boldsymbol{I}(\boldsymbol{y},\boldsymbol{y}_d)|$ *of* $\overline{\mathbf{SOP}^{\stackrel{\perp}{=}}_{\text{Clif}}}$*:*

$$F(t) := \frac{1}{\sqrt{2}^{2p}}\sum_{\boldsymbol{y},\boldsymbol{y}',\boldsymbol{y}_d} e^{2i\pi\left(P(\boldsymbol{y},\boldsymbol{y}_d)\text{-}P(\boldsymbol{y}',\boldsymbol{y}_d)\right)} |\boldsymbol{O}(\boldsymbol{y},\boldsymbol{y}_d),\boldsymbol{O}(\boldsymbol{y}',\boldsymbol{y}_d)\rangle\langle\boldsymbol{I}(\boldsymbol{y},\boldsymbol{y}_d),\boldsymbol{I}(\boldsymbol{y}',\boldsymbol{y}_d)|$$

This new functor $F$ can be seen as a simplified CPM construction, applicable only for terms that are already simplified (in the form of Lem. 5).

**Proposition 13.** *For any* $t \in \overline{\mathbf{SOP}^{\ground}_{\text{Clif}}}$, $F(t) \underset{\text{Clif+}}{\sim} \mathrm{CPM}(t)$.
*This implies* $[\![F(.)]\!] = [\![\mathrm{CPM}(.)]\!]$.

**Definition 9.** *We define a function $G$ on some morphisms of $\mathbf{SOP}_{\text{Clif}}$ that have an appropriate form. Let* $t = \frac{1}{\sqrt{2}^{2p}} \sum_{\boldsymbol{y}} e^{2i\pi P} |\boldsymbol{O}_1, \boldsymbol{O}_2\rangle\langle \boldsymbol{I}_1, \boldsymbol{I}_2|$ *with* $|\boldsymbol{O}_1| = |\boldsymbol{O}_2|$ *and* $|\boldsymbol{I}_1| = |\boldsymbol{I}_2|$. *Let us partition $\boldsymbol{y}$ into:* $\{\boldsymbol{y}_d\} := \{\boldsymbol{y}\} \setminus \mathrm{Var}(\boldsymbol{O}_1 \oplus \boldsymbol{O}_2, \boldsymbol{I}_1 \oplus \boldsymbol{I}_2)$, $\{\boldsymbol{y}_1\} := \mathrm{Var}(\boldsymbol{O}_1, \boldsymbol{I}_1) \setminus \{\boldsymbol{y}_d\}$ *and* $\{\boldsymbol{y}_2\} := (\{\boldsymbol{y}\} \setminus \{\boldsymbol{y}_1\}) \setminus \{\boldsymbol{y}_d\}$. *If* $|\boldsymbol{y}_1| = |\boldsymbol{y}_2|$ *and if there exists a unique bijection* $\delta : \{\boldsymbol{y}_2\} \to \{\boldsymbol{y}_1\}$ *such that:*
$(\boldsymbol{O}_1 \oplus \boldsymbol{O}_2, \boldsymbol{I}_1 \oplus \boldsymbol{I}_2)[\boldsymbol{y}_2 \leftarrow \delta(\boldsymbol{y}_2)] = \mathbf{0}$, *then $G(t)$ is defined, and:*

$$G(t) := \frac{1}{\sqrt{2}^p} \sum_{\boldsymbol{y}_1, \boldsymbol{y}_d} e^{-2i\pi P[\boldsymbol{y}_1 \leftarrow \mathbf{0}][\boldsymbol{y}_2 \leftarrow \delta(\boldsymbol{y}_2)]} \big( |\boldsymbol{O}_2\rangle ! \{\boldsymbol{y}_d\} \langle \boldsymbol{I}_2| \big) [\boldsymbol{y}_1 \leftarrow \mathbf{0}][\boldsymbol{y}_2 \leftarrow \delta(\boldsymbol{y}_2)]$$

The function $G$ is designed to be an inverse of $F$ for morphisms where it is defined, while at the same being impervious to some rewrite rules.

**Proposition 14.** *Let $t$ be terminal with* $\underset{\text{Clif}\,\ground}{\longrightarrow}$, *and $t'$ such that* $F(t) \overset{*}{\underset{\text{Clif}\,+}{\longrightarrow}} t'$. *Then, $G(F(t))$ and $G(t')$ exist, and $G(F(t)) = G(t')$.*

**Theorem 3.** *Let $t_1$ and $t_2$ be two morphisms of* $\mathbf{SOP}^{\ground}_{\text{Clif}}$ *such that* $[\![t_1]\!] = [\![t_2]\!]$. *If $t_1'$ and $t_2'$ are terminal such that* $t_1 \overset{*}{\underset{\text{Clif}\,\ground}{\longrightarrow}} t_1'$ *and* $t_2 \overset{*}{\underset{\text{Clif}\,\ground}{\longrightarrow}} t_2'$, *then* $t_1' = t_2'$ *up to $\alpha$-conversion.*

*Remark 2.* Interestingly, the previous proposition and theorem show that the simplification of a term of $\mathbf{SOP}^{\ground}_{\text{Clif}}$ can be operated in the "pure" setting, and then $G$ can be used to retrieve the normal form.

**Corollary 6.** *The equality of morphisms in* $\mathbf{SOP}^{\ground}_{\text{Clif}} / \underset{\text{Clif}\,\ground}{\sim}$ *is decidable in time polynomial in the size of the phase polynomial and in the combined size of the ket/bra/discarded polynomials.*
